# An in vivo animal study assessing long-term changes in hypothalamic cytokines following perinatal exposure to a chemical mixture based on Arctic maternal body burden

**DOI:** 10.1186/1476-069X-10-65

**Published:** 2011-07-11

**Authors:** Shawn Hayley, Emily Mangano, Geoffrey Crowe, Nanqin Li, Wayne J Bowers

**Affiliations:** 1 Department of Neuroscience, Carleton University, 1125 Colonel By Drive, Ottawa, K1S 5B6, Canada; 2 Environmental Health Science and Research Bureau, Health Canada, 50 Colombine Driveway, Ottawa, K1A OK9, Canada

## Abstract

**Background:**

The geographic distribution of environmental toxins is generally not uniform, with certain northern regions showing a particularly high concentration of pesticides, heavy metals and persistent organic pollutants. For instance, Northern Canadians are exposed to high levels of persistent organic pollutants like polychlorinated biphenyls (PCB), organochlorine pesticides (OCs) and methylmercury (MeHg), primarily through country foods. Previous studies have reported associations between neuronal pathology and exposure to such toxins. The present investigation assessed whether perinatal exposure (gestation and lactation) of rats to a chemical mixture (27 constituents comprised of PCBs, OCs and MeHg) based on Arctic maternal exposure profiles at concentrations near human exposure levels, would affect brain levels of several inflammatory cytokines

**Methods:**

Rats were dosed during gestation and lactation and cytokine levels were measured in the brains of offspring at five months of age. Hypothalamic cytokine protein levels were measured with a suspension-based array system and differences were determined using ANOVA and post hoc statistical tests.

**Results:**

The early life PCB treatment alone significantly elevated hypothalamic interleukin-6 (IL-6) levels in rats at five months of age to a degree comparable to that of the entire chemical mixture. Similarly, the full mixture (and to a lesser degree PCBs alone) elevated levels of the pro-inflammatory cytokine, IL-1b, as well as the anti-inflammatory cytokine, IL-10. The full mixture of chemicals also moderately increased (in an additive fashion) hypothalamic levels of the pro-inflammatory cytokines, IL-12 and tumor necrosis factor (TNF-α). Challenge with bacterial endotoxin at adulthood generally increased hypothalamic levels to such a degree that differences between the perinatally treated chemical groups were no longer detectable.

**Conclusions:**

These data suggest that exposure at critical neurodevelopmental times to environmental chemicals at concentrations and combinations reflective of those observed in vulnerable population can have enduring consequences upon cytokines that are thought to contribute to a range of pathological states. In particular, such protracted alterations in the cytokine balance within the hypothalamus would be expected to favor marked changes in neuro-immune and hormonal communication that could have profound behavioral consequences.

## Background

A wide array of substances found in the environment, including metals (e.g. lead, iron, mercury), polychlorinated biphenyls (PCBs) and pesticides can be toxic to the central nervous system (CNS). Sensitive sub-populations, such as the developing fetus, as well as elderly individuals that are already at increased risk of illness may be especially vulnerable to the neurological effects of such toxins [1-4]. Indeed, considerable epidemiological evidence indicates that prenatal or perinatal exposure to PCBs, lead, and organochlorine pesticides (OCs) can cause attention, memory and motor disturbances later in life [5-11]. A number of epidemiological studies have also demonstrated correlations between environmental pollutants and neurodegenerative disease, such as the increased incidence of Parkinson's disease in rural agricultural populations with high use of broad classes of insecticides (e.g., rotenone), herbicides (e.g., paraquat) and rodenticides [12-14].

Aboriginal people of northern Canada may be at greater risk of health problems than other populations because of higher levels of pollutants in the environment and food chain, as well as their greater reliance on country food as part of their diet. Indeed, numerous studies reported that high levels of heavy metals (particularly MeHg), PCBs, and pesticides (e.g. DDT) bioaccumulate in marine animals, fish and other wildlife [15-20]. These findings are consistent with the fact that in several indigenous northern Canadian populations (Dene, Cree and Inuit), maternal mercury and PCB levels were within the range expected to increase the risk of neurological damage and cause impairment of memory and executive functioning in their offspring [21-23].

An important but often overlooked aspect of most toxicological studies aimed at identifying health risks is the fact that assessment of the effects of a single compound can be misleading since individuals are typically exposed to multiple pollutants over time. This is particularly important when one considers the substantial evidence that environmental insults, such as pesticides, can interact to additively or even synergistically provoke neuronal damage [24-26]. To this end, we have begun to conduct studies using a mixture of pollutants (as well as the key individual constituents of the mixture; PCBs, OCs, MeHg) based on the profile of chemicals actually found in Arctic maternal Canadian populations. Gestational and lactational exposure to this Arctic chemical mixture produced blood levels in rodents that were comparable to those found in Inuit maternal blood and induced a range of dose-dependent pathological changes in offspring [27].

Although many cellular mechanisms likely contribute to the neuropathological consequences of environmental pollutants, recent evidence suggests a particular importance for neuroinflammatory factors [28,29]. For instance, pro-inflammatory cytokines, as well as the immunocompetent microglial cells, have been implicated in several neurodegenerative diseases and were reported to contribute to the neuronal damage induced by pesticides, heavy metals and other potential toxins [30-33]. Indeed, our own work has revealed that enhanced microglia and cytokine activity was associated with the neurodegenerative effects of the commonly used pesticide, paraquat [30,34].

The present study sought to evaluate the alterations of a panel of pro- and anti-inflammatory cytokines within the hypothalamus of adult rats that previously received in utero plus lactational exposure to the Arctic chemical mixture (or individual constituents). Using a perinatal exposure regimen is expected to mimic the "real life" exposure pattern of the Arctic human population. It was also of interest to determine whether perinatal exposure to these chemical agents would augment the neuroinflammatory consequences following exposure to the bacterial endotoxin, lipopolysaccharide (LPS), later in adulthood. Indeed, recent studies revealed that perinatal exposure to LPS caused a long-term elevation of TNF-α within the brain that was associated with an enhanced neuronal susceptibility to subsequent pesticide exposure in adulthood [35]. Cytokine protein levels were determined using a sensitive, laser based bead assay system that allowed us to simultaneously assess multiple cytokines. The hypothalamus was examined given the well know endocrine effects of several toxins, as well as the higher cytokine levels than most brain regions and sensitivity to immune and stressor challenges [36,37]. Ultimately, the combined prenatal chemical toxin exposure followed by adult LPS challenge should be relevant to the actual intermittent exposure to various classes of toxins (immune, chemical, organic) at critical neurodevelopmental and later times that likely occur in certain vulnerable individuals.

## Methods

### Breeding conditions

Female offspring from nulliparous female Sprague Dawley rats (Charles River Laboratories, St Constant, Quebec) were used in the present study. All animals were housed in plastic hanging cages measuring 35 (L) × 30 (W) × 16.5 (H) cm with shaved wood bedding in housing rooms maintained at 22 ± 2°C and 50 ± 10% humidity. Breeding was initiated three weeks after the animals arrived in the facility and was conducted by placing two females into each male cage and monitoring females two times daily for vaginal plugs. Once a vaginal plug was detected, the female was removed from the male cage and housed individually. The day of detection of a vaginal plug was denoted as gestation Day 0 (GD0). Beginning on gestation Day 18, dams were monitored two times daily for parturition at 08:00 and 20:00. The day of birth is denoted as postnatal Day (PND) 0. Pups were sexed on PND 1 and gender confirmed on PND 2,3 and 4. Litters were culled to eight pups on PND 4 (four males and four females where possible) by randomly selecting four males and four females from each litter to remain in the litter. Male offspring were assigned to a separate study and two female offspring per litter were assigned to the present investigation. The third female from each litter were assigned for histopathological analysis to be reported separately.

### Chemical administration procedures

Treatment procedures began on GD 1 and continued until weaning at PND 21. Specifically, pregnant dams received dosed small cookies (Teddy Graham cookies, Nabisco Ltd., Toronto, ON) with a measured volume of the appropriate dosing solution (1 ul/g body weight) in corn oil. The dosing volume added to cookies was adjusted daily based on the body weight that was collected daily. This dosing method permits precise control over dosing during gestation and lactation where food or fluid intake can vary significant. Cages were checked daily to verify that dams consumed the dosed cookies. Importantly, using this procedure, offspring were never dosed directly but rather received the toxins through placental transfer during gestation and then from the dams milk during perinatal lactation. Hence, the pups receive indirect exposure to the toxin or vehicle dosed cookies for a total of 42 days (21 days of gestation + 21 days of lactation).

Separate groups of pregnant females (N = 9-12) were dosed with either corn oil vehicle, 0.05 mg/kg/day of the full Arctic mixture, 0.01 mg/kg/day PCB, 0.02 mg/kg MeHg, or 0.03 mg/kg/day PCB+MeHg (as shown in Table 1) from GD1 to PND 21. The choice of combined PCB+MeHg was used based on previously collected data indicating that MeHg toxicity was attenuated in mixture treated animals. (Bowers, unpublished observations) This group permitted us to determine if the PCBs contributed to the reduced MeHg toxicity in mixture-treated animals. Note that the doses of the mixture components (e.g., PCBs only) were identical to the dose contained in the complete Arctic mixture.

**Table 1:** Concentrations of individual chemicals in the dosing solutions of Arctic mixture, the PCBs, and methylmercury

		Concentration (μg/ml)			
		
Chemical		Full Mix	PCB Alone	MeHg Alone	PCB+MeHg
**PCBs**	28	0.065	0.065	X	0.065

	52	0.132	0.132	X	0.132

	99	0.837	0.837	X	0.837

	101	0.120	0.120	X	0.120

	105	0.141	0.141	X	0.141

	118	0.652	0.652	X	0.652

	128	0.067	0.067	X	0.067

	138	1.956	1.956	X	1.956

	153	3.390	3.390	X	3.390

	156	0.264	0.264	X	0. 264

	170	0.541	0.541	X	0.541

	180	1.379	1.379	X	1.379

	183	0.167	0.167	X	0.167

	187	0.687	0.687	X	0.687

**OCs**	Aldrin	0.065	X	X	X

	β-BHC	0.653	X	X	X

	Cis-nonachlor	0.583	X	X	X

	p,p'-DDE	9.075	X	X	X

	p,p'-DDT	0.580	X	X	X

	Dieldrin	0.264	X	X	X

	Hexachlorobenzene	3.249	X	X	X

	Heptachlor epoxide	0.273	X	X	X

	Mirex	0.271	X	X	X

	Oxychlordane	2.086	X	X	X

	Toxaphene	1.20	X	X	X

	Trans-nonachlor	2.367	X	X	X

**MeHg**		18.918	X	18.918	18.918

**TOTAL PCB**		**10.397**	**10.397**	**X**	**10.397**

**TOTAL OCs**		**20.664**	**X**	**X**	**X**

**TOTAL all**		**49.979**	**10.397**	**18.918**	**29.316**

The PCB+MeHg dosing solution contained only PCBs and MeHg. Pregnanat females rats were dosed by applying 1 ul/g body weight on small cookies and providing these to the pregnant dams.

On PND 21, males and females were weaned and housed in same sex groups of three in standard plastic cages with ad libitum access to food and water. Male offspring were assigned to a separate study and female offspring were used in the present investigation. At PND 65, female offspring were re-housed in pairs until PND 145-147, when the animals were sacrificed by decapitation. Brains were removed and the whole hypothalamus was dissected using a rat brain matrix [37,38] and frozen in liquid nitrogen and stored at -80°C until processing.

On PND 208-212, a separate set of female litter-mates were challenged with the bacterial endotoxin, lipopolysaccharide (LPS; 48 ug/kg; i.p.) and 90 minutes later were sacrificed by decapitation and the hypothalamus was dissected and frozen for subsequent cytokine analysis (see procedures below for dissection and assay details). We have previously found that similar endotoxin doses readily provoked behavioural, neurochemical and cytokine changes within hypothalamic and stressor-sensitive limbic regions [36,39,40]. Hence, we sought to assess whether early life exposure to the Northern toxin constituents would enhance the neuroinflammatory cytokine cascade that is provoked by LPS challenge. Indeed, this situation should mimic instances where, individuals are exposed to multiple environmental contaminants and subsequently encounter typical infectious agents.

### Mixture Preparation

The contaminant profile (relative concentrations) in the mixture was based on the concentration of chemicals found in maternal blood of Inuit population in Arctic Canada [41]. Data on the concentration of the chemicals in maternal blood of Inuit women were obtained and the mean, geometric mean and median concentration in μg/l blood (lipid adjusted) were calculated. Because most pollutants included were lipophilic chemicals that are sensitive to variations in human blood lipid levels, lipid-adjusted concentrations were used in all calculations of relative contribution of lipophilic contaminants to the contaminant profile. Methylmercury was excluded from these initial relative mass concentrations because it is not lipophilic. The contribution of MeHg to the mixture was based on the MeHg: lipophilic contaminant ratio. Because a number of the chemicals had extreme values for blood levels the geometric mean blood level value was used. The median concentration of each of the chemicals was then summed to calculate the median total mass of chemicals among this population. The relative contribution (by mass) of each specific chemical was then calculated as the percent (by mass) of each chemical to the total chemical load (by mass). Chemicals that contributed more than 1% to the total mass were selected for inclusion in the mixture. This percent mass value for lipophilic chemicals then represented the percent by mass of each chemical to the overall chemical mixture and served as the basis for preparing the mixture. The final proportionate mass of each chemical in the final mixture then represented the proportionate mass of each chemical found in human blood in μg/l. Once the relative proportions of the lipophilic contaminant was established, the concentration of MeHg was calculated on the basis of the ratio of MeHg to total PCB shown in Arctic maternal blood. The final MeHg:PCB ratio was 1.8:1 in the complete mixture and accounting for 38% of the total mixture mass. Finally, prior to any dosing, the mixture was analyzed to verify the chemical concentration both internally and in an independent laboratory (Wellington laboratories, Guelph, Ontario). In both cases, these analyses confirmed the concentration in the final chemical mixture.

All of the chemicals had a purity > 99% with the exception of PCB187, which had a certified purity of 97%, and toxaphene (Cerilliant, Round Rock, Tx) and methylmercury (Aldrich Chemical Co, Milwaukee, WI), which were of technical grade. Oxychlordane was a generous gift from Julie Fillion of the Pest Management Regulatory Agency (Ottawa, ON, Canada). Sources for other chemicals were as follows: PCB 99 and 183 (AccuStandard, New Haven, CT); p,p'-DDE (Sigma-Aldrich, St Louis, MO), p,p'-DDT (Riedel-de Haën, Sigma-Aldrich Laborchemikalien, Seelze, Germany), and hexachlorobenzene (Fluka, Steinhein, Switzerland). All other chemicals were purchased from Cerilliant (Round Rock, Tx). Separate stock solutions of the PCBs, the OC pesticides, and the MeHg in corn oil were initially prepared. For the OC stock solutions, the OC chemicals (total mass 556.84 mg) were dissolved in 40 ml of spectrophotometric grade, inhibitor free diethyl ether (99.9%, Sigma-Aldrich), and then added drop-wise to 60 ml of Mazola corn oil while stirring, and the corn oil was continually stirred for a further 30 minutes in a fume hood. This solution was then evenly separated into six 20 ml scintillation vials and the diethyl ether was removed using a Savant Automatic Environment Speedvac (Model AES 2000). The complete removal of the diethyl ether was confirmed by weighing every two hours until the difference of weights of two continuous weighing was within 5 mg for each vial. The OC stock was then quantitatively transferred to a 250 ml amber bottle with fresh corn oil rinsing the vials and well mixed (final concentration = 5.273 mg/ml corn oil). The PCB stock solution was prepared in a similar way. The PCB congeners (total mass 843.14 mg) were dissolved in 25 ml of diethyl ether, and added to 80 ml Mazola corn oil. The PCB corn oil solution was evenly separated into eight 20 ml scintillation vials and the diethyl ether was removed. This PCB corn oil solution was then quantitatively transferred to a 250 ml amber bottle and the final concentration of PCB in the corn oil is 5.954 mg/ml. MeHg was prepared by dissolving 1.5167 g MeHg into 100 ml of Mazola corn oil by stirring and intermittent ultrasound sonication over two days and quantitatively transferred to a 250 ml amber bottle and the concentration of MeHg in the corn oil is 12.11 mg/ml. The dosing solution for full mixture (5.0 mg/ml) was prepared by combining 84.1 g of the OC stock solution, 37.5 g of the PCB stock solution, 33.8 g of the MeHg stock solution and 59.1 g of corn oil and then diluting this by adding 1 ml of this solution into 99 ml of corn oil. The dosing solution for the PCB+MeHg solution (1.89 mg/ml) was prepared by adding 37.5 g of the PCB stock solution with 33.8 g of the MeHg stock solution and 142.8 g of corn oil and then diluting this by adding 1 ml of this solution into 99 ml of corn oil. The dosing solution for the PCB only solution (1.04 mg/ml) was prepared by mixing 37.5 g of the PCB stock solution with 172.2 g of corn oil and then diluting this by adding 1 ml of this solution into 99 ml of corn oil. The dosing solution for the MeHg only solution (1.89 mg/ml) was prepared by mixing 33.8 g of the MeHg stock solution with 180.9 g of corn oil and then diluting this by adding 1 ml of this solution into 99 ml of corn oil. The concentration of each chemical in each dosing solutions for is shown in Table 1.

### Brain dissection procedures

Following decapitation, rat brains were placed in a stainless steel brain blocker and a series of coronal sections (1.0 mm thick) were produced. As already indicated we chose to focus on the hypothalamus given that this region has higher than normal levels of most cytokines and the fact that most research indicates a critical role for hypothalamic functioning in cytokine induced neuronal alterations. The hypothalamus was then rapidly dissected from the two coronal sections containing this structure. Brain tissue was then flash frozen in liquid nitrogen and stored at -80°C until assayed. Thereafter, brain tissue was homogenized and centrifuged at 6000 RPM for 10 min at 4°C, after which 50 μl of the supernatant was collected for use in the Luminex analysis.

### Multiplex Luminex determination of brain cytokine levels

The Luminex 100 (Luminex Corp., Austin, TX) is a suspension-based bead array system that can detect up to 100 different analytes in a single 50 μl sample. Sets of microspheres (5.6 μm beads) are internally dyed with different ratios of fluorophores, each conjugated to a different capture probe (cytokine specific antibody). Following incubation, a classification laser identifies the particular cytokine bound and a second reporter laser quantifies the signal present. We utilized a custom multiple cytokine detection kit (Beadlyte Mouse Multi-Cytokine Detection System, Upstate Cell Signalling Solutions) to detect levels of IL-1b, IL-2, IL-4, IL-6, IL-10, IL-12, IFN-g and TNF-α.

To prepare standards for Luminex analysis, 5000 pg of Multi-Cytokine 2 standard was re-suspended in 1 ml serum diluent and vortexed at a medium speed for 15 s, following which serial dilutions were prepared. After 25 μl of Beadlyte Cytokine Assay Buffer was added to the wells, plates were vortexed and a vacuum manifold applied to remove excess liquid. Subsequently, 25 μl of serum diluent and 25 μl of sample were added to each well. Following 20 min incubation on a shaker, the anti-mouse multi-cytokine beads were vortexed, sonicated and 25 μl of the bead solution added to the wells. After a brief vortex, plates were then incubated overnight at 4°C. Thereafter, samples were re-suspended in 50 μl of Beadlyte Cytokine Assay Buffer and the vortex and washing procedures repeated. Finally, 25 μl of biotin conjugated cytokine beads were added for 90 min incubation in the dark. Just prior to the end of the incubation period, the Beadlyte Streptavidin-PE was diluted (1:25) and 25 μl was added to each well for the final 30 min incubation. The assay was then halted using 25 μl of Beadlyte Stop Solution. Filter plates were then read in a Luminex 100 instrument, fitted with a five-parameter logistic regression curve using QT Masterplex software (MiraiBio, Hitachi, CA) [42,30].

### Statistical Analyses

All data were analyzed using ANOVA followed by Tukey's post hoc comparisons where appropriate. In effect, we assessed whether 1. perinatal treatment with PCBs or MeHg induced cytokine changes, relative to vehicle (Veh) only treated group, and 2. whether the combined PCB + MeHg or full Arctic chemical mixture promoted further cytokine elevations greater than their individual effects. In our second study, female litter-mates received the same treatments except that all rats were administered LPS on PND 208-212 in order to determine if the cytokine response to LPS was modified by previous perinatal exposure to the chemicals. Hence, the first study examined basal cytokine levels at PND 145-147, while the second study used litter-mates that were administered LPS 90 min before sacrifice at PND 208-212. Data were evaluated using a StatView (version 6.0) statistical software package available from the SAS Institute, Inc.

## Results

The cytokines IL-2 and IL-4 were below detection levels for most animals and likewise, substantial variability of IFN-g precluded detection of any reliable between group differences. Hence, these cytokines are not further discussed.

**Figure 1:**
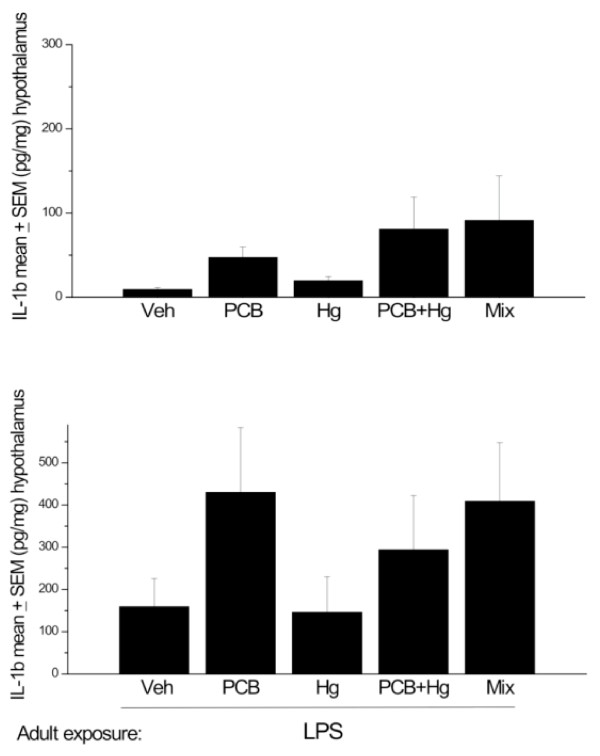
**Hypothalamic IL-1β changes in offspring at PND 145-147 (A) and in animals at PND 208-212 that received LPS immediately before sacrifice (B)**. All animals were exposed to the chemical mixture, PCBs, MeHg or MeHg+PCBs during gestation and lactation.. n = 8-10

The ANOVA for hypothalamic IL-1b levels just missed significance with regards to the effects of perinatal chemical exposure F (4, 33) = 2. 52, p = 0.07). Indeed, as shown in Figure 1a there was a definite trend towards elevated levels of the cytokine among rats that received the PCB + MeHg or full Arctic mixture treatments. Although IL-1b levels were (not surprisingly) appreciably greater overall in the second study that involved adult LPS administration (compared to non-LPS treated rats of study 1), no significant differences were apparent with regards to the Arctic chemical treatments (see Figure 1b). Although variability within the treatment groups prevented finding statistical significance, it is important to underscore that the IL-1b levels in the PCB + LPS and full mixture + LPS treatment conditions were elevated by ~2.5 times that of the vehicle + LPS group.

**Figure 2:**
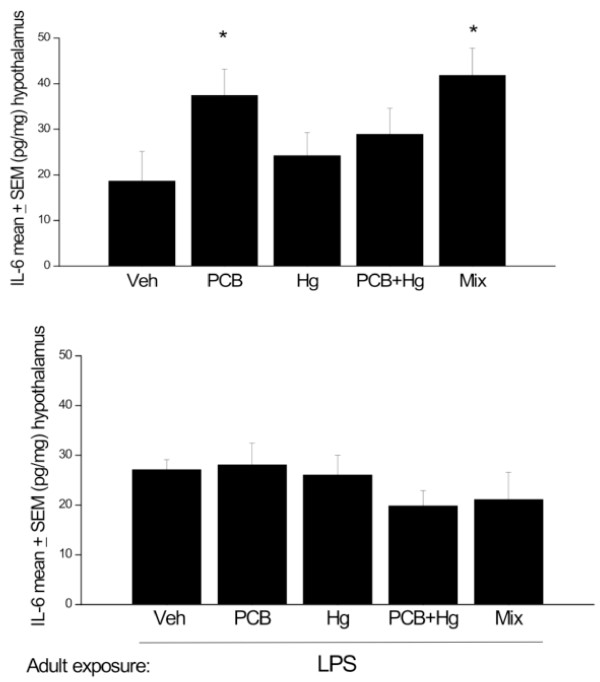
**Hypothalamic IL-6 changes at PND 145-147 (A) and in animals at PND 208-212 that received LPS immediately before sacrifice (B)**. All animals were exposed to the chemical mixture, PCBs, MeHg or MeHg+PCBs during gestation and lactation.. n = 8-10. * p < 0.05, relative to Veh or Hg treated groups.

Perinatal exposure to the Arctic chemicals did significantly affect hypothalamic IL-6 levels F (4, 33) = 2. 79, p < 0.05. Indeed, the follow up comparisons revealed that PCB alone, as well as the full mixture of chemicals increased IL-6 levels above that of rats treated with vehicle or MeHg (p < 0.05; see Figure 2a). In contrast, no significant differences in hypothalamic IL-6 levels were evident among the perinatal chemical treatment groups that received an LPS injection in adulthood (Figure 2b).

**Figure 3:**
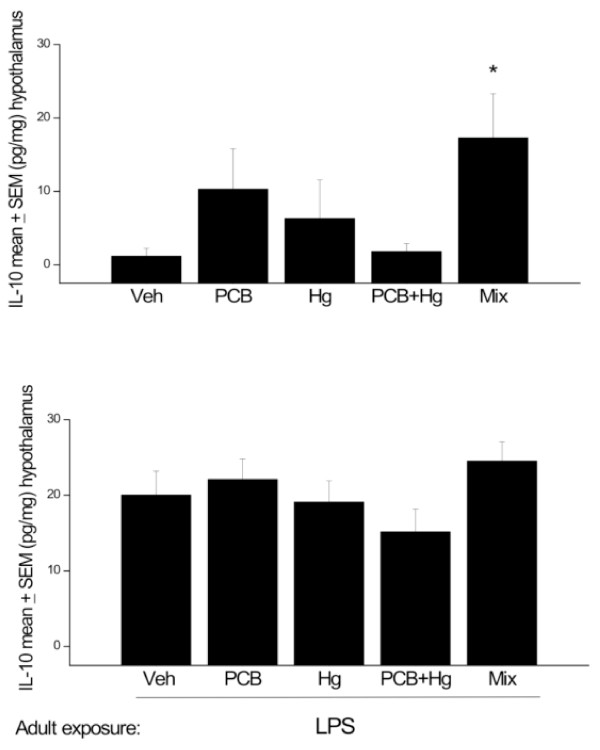
**Hypothalamic IL-10 changes at PND 145-147 (A) and in animals at PND 208-212 that received LPS immediately before sacrifice (B)**. All animals were exposed to the chemical mixture, PCBs, MeHg or MeHg+PCBs during gestation and lactation. n = 8-10. * p < 0.05, relative to Veh and PCB + Hg treated groups.

Perinatal treatment with the Arctic chemicals provoked significant differences in hypothalamic IL-10 levels F (4, 33) = 2.32, p < 0.05. Interestingly, only rats that received the full Arctic chemical mixture displayed hypothalamic IL-10 levels that exceeded that of vehicle treated animals, as well as those exposed to MeHg or MeHg + PCB (p < 0.05; Figure 3a). Once again, no significant differences were observed among the chemical treated groups that were administered LPS in adulthood (P > 0.05; see Figure 3b).

**Figure 4:**
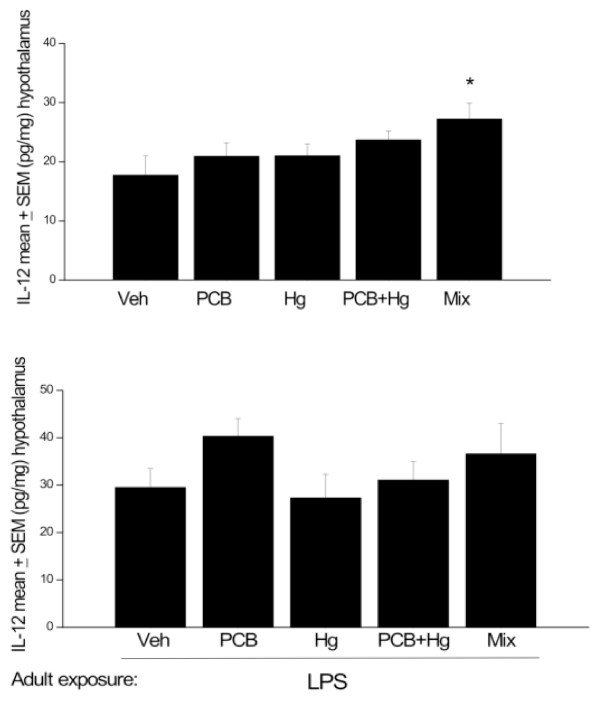
**Hypothalamic IL-12 changes at PND 145-147 (A) and in animals at PND 208-212 that received LPS immediately before sacrifice (B)**. All animals were exposed to the chemical mixture, PCBs, MeHg or MeHg+PCBs during gestation and lactation. n = 8-10. * p < 0.05, relative to Veh treated group.

**Figure 5:**
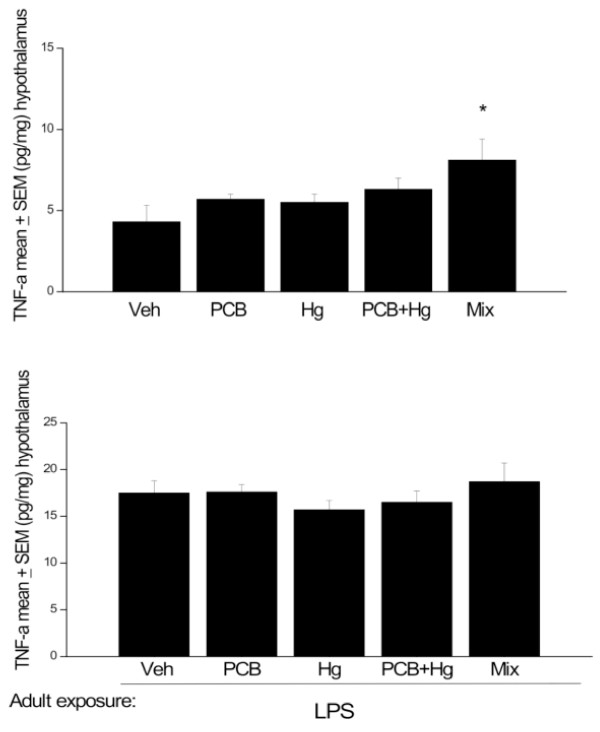
**Hypothalamic TNF-α changes at PND 145-147 (A) and in animals at PND 208-212 that received LPS immediately before sacrifice (B)**. All animals were exposed to the chemical mixture, PCBs, MeHg or MeHg+PCBs during gestation and lactation. n = 8-10. * p < 0.05, relative to Veh treated group.

No significant differences in hypothalamic IL-12 or TNF-α levels were evident between the perinatal chemical treatments. However, it is worthwhile to note the substantial trends towards increased levels of these cytokines as a function of combined administration of the Arctic chemicals. In particular, IL-12 and TNF-α levels were approximately 40% higher in rats exposed to the full Arctic mixture, relative to those that received vehicle (see Figures 4a &5a). Thus, we conducted post hoc analyses based on our hypothesis that these pro-inflammatory cytokine would be elevated by the early life chemical treatments. In this regard, rats that received the full mixture perinatally displayed significantly increased IL-12 and TNF-α concentrations, relative to vehicle treated controls (p < 0.05). Again, although LPS treated rats had greater overall IL-12 and TNF-α levels compared to those that did not receive the endotoxin, there were no detectable differences between perinatal treatment groups (Figures 4b &5b).

## Discussion

Apart from the influence of genetic differences, certain populations of individuals might be at higher risk of CNS disturbances owing to increased exposure associated with food intake patterns related to lifestyle. In this regard, Arctic populations have higher than average (compared populations living in Southern Canadian locations) concentrations of several toxins (including MeHg, pesticides and PCBs), largely owing traditional diets consisting of wildlife that bioaccumulate these environmental chemicals. The high levels of metals, PCBs, and pesticides (e.g. DDT) consumed from fish and other species of northern Canada [16-18] would be expected to readily penetrate multiple organs and eventually enter the brain.

We presently report that in utero + gestational exposure to a mixture of chemical contaminants (MeHg, pesticides, PCBs), based on blood contaminant profiles in Northern Canadian Inuit Arctic mothers, produced long term elevations of several cytokines within the hypothalamus of female rats. These effects on hypothalamic brain cytokines were evident in adulthood long after dosing ceased (about 120 days after dosing) and were observed when toxin exposure produced blood levels in rat dams near those of humans. Indeed, analysis of tissue residue data from previous studies using identical dosing procedures showed that blood levels in rat mothers were comparable to those of Arctic human mothers [27]. Yet, one should still exercise caution when extrapolating between human and rat samples. While early life exposure to MeHg had little impact on cytokines in the present study, the PCBs and the full mixture (containing PCBs, OCs and MeHg) both elevated basal cytokine levels when assessed at five months of age. Although systemic exposure to LPS at five months of age increased most hypothalamic cytokines, prior developmental exposure to the contaminants did not alter the impact of LPS on adult cytokine levels. Thus, developmental exposure to realistic levels of environmental chemicals provoked long-term inflammatory cytokine elevations within the brain but did not sensitize animals to the impact of endotoxin exposure at adulthood.

These findings are consistent with the evidence indicating that exposure to environmental toxins during neurodevelopment can influence central nervous system (CNS) functioning long after exposure has occurred. For instance, gestational exposure to PCBs, mercury, lead, and organic pollutants has been associated with later cognitive disturbances in infants and children and may contribute to disorders of attention and activity [9,43-45]. Yet, such cognitive effects are generally mediated by hippocampal and cortical brain regions, whereas hypothalamic brain changes (as observed in the present investigation) are typically associated with stress responses and hormonal output. Indeed, a plethora of data indicates that psychological and immunological (particularly LPS) stressors promote marked hypothalamic neurochemical alterations, often coupled with signs of sickness (e.g. fever, piloerection, ptosis, curled body posture) or depressive-like symptoms, such as anhedonia [38,40,46-49]. Similarly, we and others have reported that a range of stressful conditions (particularly psychosocial stressors stemming from changes in housing conditions), cytokines (including IL-1b, TNF-α and IFN-α) and immune agents that mimic bacterial or viral infections (LPS and poly I:C, respectively) increase hypothalamic cytokine expression and promote microglial-dependent neuroinflammatory activity [39,50-53].

Although scant data exists regarding the impact of chemical toxins and hypothalamic functioning, one recent report did indicate that the pesticide, dieldrin, increased hypothalamic expression of an array of genes that are known to be responsible for oxidative functions and cell survival [54]. Similarly, MeHg was found to reduce hypothalamic dopamine levels and induce anxiety-like effects in exposed fish [55]. Hypothalamic and limbic brain circuits, along with a shift towards increased production of pro-inflammatory Th1 cytokines, were even posited to be responsible for the sickness symptoms provoked by smells associated with previous chemical toxin exposure [56].

Further rationale for focusing upon the hypothalamus (besides it being a key stress integrative brain region that is known to express a higher level of cytokines than most brain regions), stems from the substantial evidence showing that several pesticides and PCBs have well known endocrine disruption effects and have been reported to affect HPA and immune functioning. For instance, systemic administration of the PCB mixture, Aroclor 1248, altered glucocorticoid levels and the mitogenic response of peripheral immune cells [57]. When an alternate PCB mixture (Aroclor 1254) was orally administered to female monkeys, dose-dependent alterations of T cell activity and antibody production were observed [58,59]. Intriguingly, perinatal exposure to PCB congers 126 and 153 (as in the present study) appeared to sensitize the HPA axis, such that a much greater and prolonged cortisol response was evident with mild stress application at nine months of age [60]. Similar to the PCBs, several different classes of pesticides were reported to affect HPA functioning in a number of different species, including male and female rats, as well as bears and fish [61-64]. Although scant data exist for MeHg, one recent study did report that Beluga sturgeon fed MeHg rich diets displayed elevated cortisol and glucose levels [65].

While the blood levels of contaminant in these animals were not available, we have conducted previous studies using identical dosing methodology and have shown that this dose of the mixture produces blood levels of PCB and OC pesticides in rat dams that are comparable to maternal blood levels in Canadian Arctic human population [27] Table 1. Other studies using the same mixture and dosing regimen has shown that PCBs and MeHg can both alter cerebellar gene expression patterns [66,67]. Taken together with the present results, the available evidence suggests that, at exposure levels relevant to human populations, the chemical mixture likely affects neurodevelopmental processes and has long-term consequences upon cytokines that are known to fundamentally shape neuroinflammatory functioning.

Gestational and lactational transfer of environmental toxins would be expected to place the developing fetus or young offspring at risk. These would be especially evident during *in utero *and perinatal stages, when neuronal migration and synaptic pruning are occurring, neurons are especially sensitive to perturbations caused by environmental agents. At the same time, biological detoxification systems involved in metabolism and clearance of toxic substances are not fully developed in fetuses, infants and young children [68,69]. Indeed, it is likely of particular importance that toxin exposure in the present investigation occurred during times of rapid neural development, when the blood-brain-barrier (BBB) is not fully functional and the brain is exquisitely sensitive to toxic chemicals that can affect neuronal migration and differentiation, as well as synapse formation [70,71]. Some of these same chemicals, including MeHg and the various pesticides, can cause deficits in BBB functioning, evident as a long term increased permeability [72-75]. Hence, the protracted hypothalamic cytokine changes presently observed could conceivably have stemmed from deficiencies in BBB functioning induced by the Arctic chemicals, resulting in enhanced infiltration of peripheral immune cells. Yet, it is important to consider that some aspects of the hypothalamus (median eminence) actually lack a fully functional BBB and may facilitate penetration of the toxins. Besides any effects of peripheral immune cells, it seems likely that the chemical insults could have directly affected central glial activity, as has been observed following bacterial endotoxin challenge [76], thereby promoting local cytokine production [77].

One of the primary mechanisms through which toxins may promote CNS pathology is by inducing inflammatory immune factors. Indeed, neurodegeneration and CNS pathology in general, often have a prominent neuroinflammatory component, which is typically characterized by excessive microglial activation and accumulation of pro-inflammatory cytokines and oxidative factors [35,78-80]. Similarly, pesticides have been reported to increase superoxide production from circulating neutrophils, as well as promote cortical astrocyte expression and induce the expression of the pro-inflammatory cytokines IL-6, IL-8 and IFN-g [66,80-82]. Our own work has also shown that the acute adult exposure to the pesticide, paraquat, provoked neuroinflammatory changes, including an elevation of microglial cell reactivity that was closely tied to the neuronal loss provoked by the pesticide [30]. The current results further show that exposure to a combination of environmental pollutants containing OCs, when given at realistic concentration/ratios, increase hypothalamic IL-6 and IL-10, and to a certain degree, IL-1b, IL-12 and TNF-α concentrations.

The lack of statistically significant differences between the Arctic chemical treated groups that received LPS in adulthood was somewhat surprising. Indeed, it was reported that early life exposure to LPS promoted an enhanced neurodegenerative effect, coupled with increased central TNF-α levels, upon exposure to the pesticide, rotenone, later in life [83]. However, the failure to presently detect cytokine differences in response to the acute adult LPS challenge likely stems from a ceiling effect. In fact, the endotoxin did generally augment most cytokines in all groups (relative to the endotoxin naive rats of the initial study) and such an effect might have made it especially difficult to detect any subtle effects of the early life chemical treatments. Along these lines, there was a definite trend of increased hypothalamic IL-1b levels in the PCB and full Arctic mixture perinatally treated mice that received LPS in adulthood. The variability in the response to LPS apparent in these mice suggests that some animals were "responders" and some "non-responders" to the early life chemical priming. Future studies aimed at better characterizing this effect would benefit from assessing the impact of a variety of LPS doses. Along with not having a dose-response for LPS, another caveat of this second study is the lack of a "pure" control group (owing to the availability of animals) that did not receive LPS.

The cytokine changes observed within the hypothalamus could have substantial behavioral implications. For instance, IL-1b, IL-6 and TNF-α have well documented sickness effects (e.g. ptosis, piloerection, curled body posture) that are related to hypothalamic neurochemical activity [37,84,85]. These inflammatory cytokines have also been implicated in a number of clinical conditions involving a primary component of fatigue or malaise, including chronic fatigue syndrome and multiple chemical sensitivity [86,87]. In fact, disturbances of hypothalamic neuroendocrine activity and elevations of brain cytokines which are evident following challenge with the viral mimic, poly I:C (double stranded RNA), have been proposed to be common mechanisms leading to chronic fatigue and sickness [88,89]. Interestingly, pesticide exposure has likewise been implicated in multiple chemical sensitivity syndromes and general sickness symptoms [90,91]. We have also reported that the pesticide, paraquat, induced behavioral changes reminiscent of Parkinson's disease and depression [34]. In effect, it is possible that the present hypothalamic cytokine changes induced by early life chemical treatments could have important consequences for behavioral and neuroendocrine functioning.

It is unclear whether the enduring CNS cytokine alterations provoked by the perinatal chemical treatments (observed months after exposure) stemmed from cumulative/progressive time-dependent effects or a long lasting more acute impact of these treatments. In any case, it is interesting to note that several studies have indicated that stressor exposure had neurochemical effects that increased with the passage of time [92,93]. Our own work likewise demonstrated that the cytokine, TNF-α, time-dependently, sensitized CNS processes, such that re-exposure to the cytokine one month following a previous single injection induced greatly augmented behavioral (sickness symptoms), corticoid and central monoamine (NE within the hypothalamus) changes [38,94]. Regardless of the mechanisms responsible for the central cytokine variations, these immunotransmitters are ultimately able to act upon their receptors (found predominately on glial cells and to a lesser degree neurons) to induce the activation of JAK-STAT (IL-6, IL-10 and IL-12) and NFkB (IL-1b, TNF-a) signaling pathways. While these signaling pathways mediate the anti-tumor and immunological functions of cytokines in the periphery, increasing evidence has also indicated an important role in a range of neurological conditions ranging from depression to Parkinson's and Alzheimer's disease [95-97].

## Conclusions

The present investigation revealed that administration of a mixture of environmental toxins at environmentally-relevant doses induced long term elevations of cytokine protein concentrations within the hypothalamus. This is important in light of the fact that most experimental studies to date have involved administration of high levels of single chemicals that do not necessarily reflect the actual human exposures or human body burden. Indeed, it might be deceiving to evaluate toxins in isolation given that certain combinations of heavy metals and pesticides were found synergistically provoke numerous histopathological consequences (e.g. conformational changes in alpha-synuclein) and oxidative stress induced neurodegeneration [31]. Moreover, the current cytokine changes were evident in adult rats following indirect exposure (through placental transfer or from breast milk) during neurodevelopmentally sensitive times (pre- and perinatal exposure). Given the substantial data suggesting that cytokines markedly influence neurotransmission and neuronal survival, the present findings support an involvement of environmental contaminants in the development of neurological or psychiatric disturbances.

## List of Abbreviations

PCB: polychlorinated biphenyls; OC: organochloride pesticide; MeHg: methylmercury; IL-6: interleukin-6; TNF-α: tumor necrosis factor-α; CNS: central nervous system; LPS: lipopolysaccharide; GD0: gestation day 0; PND: postnatal day

## Competing interests

The authors declare that they have no competing interests.

## Authors' contributions

SH and WJB wrote the manuscript and designed the studies. EM and GC conducted the cytokine analyses. NL was involved in processing and experimentally manipulating the rats. All authors read and approved the final manuscript.
